# Identification of Molecular Markers Associated With the Pathophysiology and Treatment of Lupus Nephritis Based on Integrated Transcriptome Analysis

**DOI:** 10.3389/fgene.2020.583629

**Published:** 2020-12-15

**Authors:** Menghui Yao, Congcong Gao, Chunyi Zhang, Xueqi Di, Wenfang Liang, Wenbo Sun, Qianqian Wang, Zhaohui Zheng

**Affiliations:** Department of Rheumatology, The First Affiliated Hospital of Zhengzhou University, Zhengzhou, China

**Keywords:** systemic lupus erythematosus, lupus nephritis, bioinformatics, differentially expressed genes, WGCNA

## Abstract

Lupus nephritis (LN) is a well-known complication of systemic lupus erythematosus and is its leading cause of morbidity and mortality. Our study aimed to identify the molecular markers associated with the pathophysiology and treatment of LN. The renal tissue gene expression profiles of LN patients in the GSE32591 dataset were downloaded as a discovery cohort from the Gene Expression Omnibus. Differentially expressed genes (DEGs) were identified; weighted gene co-expression network analysis (WGCNA) was used to identify the co-expression modules of DEGs; and gene function enrichment analysis, molecular crosstalk analysis, and immune cell infiltration analysis were performed to explore the pathophysiological changes in glomeruli and tubulointerstitia of LN patients. The crosstalk genes were validated in another RNA-sequencing cohort. DEGs common in RNA-sequencing dataset and GSE32591 were uploaded to the Connectivity Map (CMap) database to find prospective LN-related drugs. Molecular docking was used to verify the targeting association between candidate small molecular compounds and the potential target. In all, 420 DEGs were identified; five modules and two modules associated with LN were extracted in glomeruli and tubulointerstitia, respectively. Functional enrichment analysis showed that type I interferon (IFN) response was highly active, and some biological processes such as metabolism, detoxification, and ion transport were impaired in LN. Gene transcription in glomeruli and tubulointerstitia might affect each other, and some crosstalk genes, such as *IRF7*, *HLA-DRA*, *ISG15*, *PSMB8*, and *IFITM3*, play important roles in this process. Immune cell infiltration analysis revealed that monocytes and macrophages were increased in glomeruli and tubulointerstitia, respectively. CMap analysis identified proscillaridin as a possible drug to treat LN. Molecular docking showed proscillaridin forms four hydrogen bonds with the SH2 domain of signal transducer and activator of transcription 1 (STAT1). The findings of our study may shed light on the pathophysiology of LN and provide potential therapeutic targets for LN.

## Introduction

Systemic lupus erythematosus (SLE) is an autoimmune disease involving multiple organs and systems, and its pathophysiology remains unclear (Mu et al., 2015). Lupus nephritis (LN) is a well-known complication of SLE; about 80% of children and 40% of adults are affected by LN (Brunner et al., 2008), which is the leading cause of morbidity and mortality in SLE patients. We performed a retrospective study for 491 LN patients in China and found that the cumulative probability of survival at 10 and 20 years are 77 and 45%, respectively (Zheng et al., 2012). At present, for the treatment of SLE and LN, most clinicians use high-dose glucocorticoids and immunosuppressants to induce remission, followed by long-term maintenance with small doses. However, only 30–50% of the patients achieve remission, and 10–20% of LN patients progress to end-stage renal disease (ESRD) (Maria and Davidson, 2020). Therefore, the treatment and prognosis of LN are generally not optimistic. It is necessary to strengthen the study of its pathophysiology further and find new treatment methods to improve the survival rates of patients with LN.

In recent years, the combination of molecular biology and information technology has led to the emergence of bioinformatics (Li et al., 2018), which has been used to reinterpret disease at the gene level and has revealed many clinical markers that may be used to diagnose disease or evaluate prognosis, especially in cancer (Zhang et al., 2018). However, there are few studies on bioinformatics in LN. Although the etiology of LN remains uncertain, it is strongly believed that the incidence of LN is associated with genomic and epigenomic mechanisms (Kwon et al., 2019). The various gene expression profiles and their regulatory mechanisms in LN remain to be illuminated.

Here, we obtained the differentially expressed genes (DEGs) of 32 LN renal tissues and 15 healthy renal tissues from the GSE32591 dataset. Functional enrichment analysis, weighted gene co-expression network analysis (WGCNA), molecular crosstalk analysis, and immune cell infiltration analysis were performed to explore the pathophysiological changes in glomeruli and tubulointerstitia of LN patients. The crosstalk genes were then validated in another cohort. Moreover, the DEGs common in an RNA-sequencing dataset and GSE32591 were uploaded to the Connectivity Map (CMap) database to find LN-related drugs. Molecular docking was used to verify the association between candidate small molecular compounds and their potential targets. The analysis of DEGs may shed light on the pathophysiology of LN and provide potential biomarkers for its treatment.

## Materials and Methods

### Subjects and Samples

Six renal tissues were obtained from biopsies of three untreated patients with LN and three patients with renal cancer from the First Affiliated Hospital of Zhengzhou University. The diagnosis of patients with LN met the 1997 American Rheumatology Association SLE Classification Criteria and international renal pathology criteria. Healthy renal tissues at least 5 cm from the tumor were taken for controls, and their unaffected status was confirmed by microscopic examination. This study was approved by the Ethical Committee of the First Affiliated Hospital of Zhengzhou University (2018-KY-22), and informed consent was obtained from the patients.

### Next-Generation Sequencing

Total RNA was extracted from the renal tissues using the TRIzol LS Reagent (Invitrogen, CA, United States). After total RNA quality check, the rRNA was removed using the Ribo-ZeroTM rRNA removal kit (Illumina, CA, United States), and purification and fragmentation of RNA were performed at the same time (the fragment length was between 100 and 300 bp to facilitate sequencing). First-strand cDNA was synthesized via reverse transcription, followed by second-strand cDNA synthesis. After terminal repair and purification, the cDNA library was amplified through PCR. Finally, samples were sequenced using a 2 × 150 base paired-end configuration with the Illumina Hiseq 2500 (Illumina, CA, United States).

### Gene Expression Omnibus Data Preprocessing

The renal tissue gene expression profiles of GSE32591 from LN patients and healthy controls were downloaded from the Gene Expression Omnibus (GEO) database. GSE32591 is a microarray dataset generated by the Affymetrix GeneChip Human Genome HG-U133A Custom CDF (Berthier et al., 2012). It included 32 patients with SLE and LN and 15 healthy controls. Then, the annotation document of corresponding platforms was used to annotate the gene expression profiling in each dataset. Finally, the matrix with row names as sample names and column names as gene symbols was obtained for subsequent analysis.

### Differentially Expressed Gene Analysis

For GSE32591, the DEGs in glomeruli and tubulointerstitia were defined by *p* < 0.05 and log_2_| fold change| > 1.0 using the “limma” package in R software 4.0.0. All the DEGs in glomeruli and tubulointerstitia were defined as total DEGs in GSE32591. For RNA-sequencing data, Deseq2 software was used to analyze the DEGs by comparing the case and control groups. The DEGs were defined by *p* < 0.05 and log_2_| fold change| > 1.0.

### Weighted Gene Co-expression Network Analysis

To explore the function of the DEGs more accurately, we identified the co-expression modules in glomeruli and tubulointerstitia using WGCNA, which is an algorithm that can specially screen genes related to the clinical traits and obtain co-expression modules with high biological significance (Langfelder and Horvath, 2008). For glomeruli, to obtain a sufficient number of genes for WGCNA analysis, the genes were ranked by their log2| fold change| value. Finally, the genes with log_2_| fold change| > 0.589 (| fold change| > 1.5) and *p* < 0.05 were selected from the final ranked gene list. For the tubulointerstitia, the genes with log_2_| fold change| > 0.380 (| fold change| > 1.3) and *p* < 0.05 were selected. The WGCNA was performed using the R package “WGCNA” (Langfelder and Horvath, 2008). First, the appropriate soft powers β was selected according to the standard of scale-free network using the algorithm “pickSoftThreshold.” Second, the adjacency coefficient a*_*ij*_* was calculated by the formula: a*_*ij*_* = | S*_*ij*_*| ^β^. The S*_*ij*_* was the Pearson correlation coefficient of gene *i* and gene *j*, β represents soft powers value. Third, a topological overlap matrix (TOM) and the corresponding dissimilarity (1-TOM) were calculated according to the adjacency coefficient. Then, a hierarchical clustering dendrogram built based on 1-TOM matrix was used to divide co-expressed genes into different modules. Fourth, the module eigengene (ME) that represented the expression patterns of each module was calculated and performed a Pearson correlation analysis with the clinical trait to obtain the modules that were significantly associated with LN.

In this study, the soft threshold was defined as 12 in WGCNA analysis of glomeruli and 18 in WGCNA analysis of tubulointerstitia. The other parameters were the following: minModuleSize = 20, networkType = “unsigned,” deepSplit = 2, and mergeCutHeight = 0.25.

### Functional Enrichment Analysis

Gene Ontology (GO) analysis was used to describe the attributes of genes and gene products, including biological process (BP), molecular function (MF), and cellular component (CC). The Kyoto Encyclopedia of Genes and Genomes (KEGG) pathways enrichment analysis was used to obtain pathways at the gene level.

For the co-expression modules obtained from the WGCNA, we focused on the DEGs with log_2_| fold change| > 1 and *p* < 0.05 due to their significant changes and performed the GO and KEGG analyses on DEGs using DAVID^1^. The results of the GO analysis related to BP and KEGG pathways were focused, and the *p*-value represented the significance of the GO terms and pathways; the smaller the *p*-value, the higher the significance.

### Molecular Crosstalk Analysis Between Glomeruli and Tubulointerstitia

As the glomeruli and tubules are closely related anatomically, we wanted to know whether the DEGs in the glomeruli and tubulointerstitia can influence each other. First, we extracted the gene expression data of DEGs from the modules identified from WGCNA and reconstructed the matrices with row names as sample names and column names as DEG symbols. Second, to obtain the correlation among these matrices, we used the principal component analysis (PCA) in SPSS 25.0 to obtain the first principal component of each matrix. Pearson correlation analysis was used to calculate the correlation between these first principal components. The whole analysis process is similar to the WGCNA “relating modules to clinical trait” analysis. Third, to further explore the mechanism of interaction between the glomerulus and tubulointerstitia, we selected the hub genes in each first principal component of matrices based on the following standards: (a) the eigengene connectivity (kME) of genes in modules > 0.9; and (b) the correlation coefficient with the first principal component in factor loading matrix > 0.8. Then, we used the Search Tool for the Retrieval of Interacting Genes (STRING) database to construct a protein–protein interaction (PPI) network of the hub genes at the protein level. We focused on the interaction between the hub genes located in different modules. The hub genes with the highest degree in the network were defined as crosstalk genes.

### Immune Cell Infiltration Analysis

The CIBERSORT algorithm is an analytical tool used to estimate the proportion of various types of immune cells in complex tissues (such as large solid tumors) (Ali et al., 2016). Panousis et al. (2019) have successfully used this algorithm to estimate the proportion of blood immune cell subsets for SLE patients. Therefore, we uploaded the gene expression data of glomeruli and tubulointerstitia to the CIBERSORT website^2^ and obtained the landscapes of immune cells in these tissues, which encompassed T cells, B cells, monocytes, eosinophils, natural killer (NK) cells, macrophages, plasma cells, neutrophils, dendritic cells, and mast cells. Wilcoxon rank sum test was used to compare the proportion of immune cells between LN renal tissues and healthy renal tissues; *p* < 0.05 was considered significant. Pearson correlation was used to evaluate the correlation between the interferon (IFN)-induced genes and immune cells with significantly different proportions.

### Validation of Crosstalk Genes

Next-generation sequencing (NGS) technology has developed rapidly in the past decade. It has great advantages for discovering unknown transcripts and comparing alternative splicing microarrays (Levy and Myers, 2016). Our team has performed deep sequencing of three cases of LN renal tissues and normal renal tissues and obtained a large number of DEGs. Therefore, we used the RNA-sequencing dataset to further validate the expression levels of crosstalk genes according to their fold change value.

### Connectivity Map Analysis and Molecular Docking

The CMap database is a database of drug-related gene expression profiles, and it consists of a large amount of genome-wide transcriptional expression data of cell lines treated with small molecular compounds to reveal the correlation among genes, diseases, and drugs (Lamb, 2007). Based on the gene expression profiles, researchers could quickly find the drugs with high relevance to diseases.

To improve the accuracy of drug screening further, we selected the common DEGs that had the same expression trend in both GSE32591 and RNA-sequencing dataset. Then, the common DEGs were converted to probe number HG133A through Affymetrix^3^. The prober numbers of upregulated genes and downregulated genes were transferred into the CMap website for analysis. The *p* < 0.05 and Enrichment < 0 indicated that the changes in the gene expression profiles caused by drugs were opposite to those caused by diseases, and these drugs might have a therapeutic effect.

Molecular docking was performed using the Swissdock website to explore whether there was a targeting association between candidate small molecular compounds and DEGs (Grosdidier et al., 2011). The UCSF Chimera software 1.14 was used to visualize the binding interactions between small molecular compounds with three-dimensional (3D) models of the target.

### Statistical Analysis

The data in this article were collated from two independent experiments. SPSS 25.0 and R software 4.0.0 were used for statistical analysis; *p* < 0.05 was considered statistically significant.

## Results

### The Expression Profile of Differentially Expressed Genes in GSE32591

**FIGURE 1:**
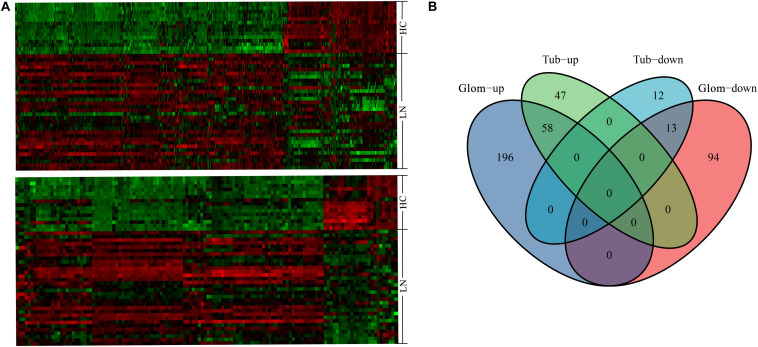
The hierarchical clustering heat maps and Venn diagrams. **(A)** The heat map above represents the differentially expressed genes (DEGs) in glomeruli; the heat map below represents the DEGs in tubulointerstitia; red represents upregulation and green represents downregulation. **(B)** The Venn diagram of the upregulated genes and downregulated genes in glomeruli and tubulointerstitia. LN, lupus nephritis; HCs, healthy controls; Glom, glomeruli; Tub, tubulointerstitia.

From the GSE32591 dataset, 361 DEGs were identified in glomeruli, including 254 upregulated genes and 107 downregulated genes. In addition, 130 DEGs were identified in tubulointerstitia, including 105 upregulated genes and 25 downregulated genes. Hierarchical clustering heat map was used to reveal the differences in the expressions of the DEGs between LN and control groups (Figure 1A). Among these DEGs, 58 genes were upregulated and 13 genes were downregulated in both glomeruli and tubulointerstitia (Figure 1B). In all, there were 420 DEGs in GSE32591, including 301 upregulated genes and 119 downregulated genes. Furthermore, the DEGs in the RNA-sequencing dataset were also identified. There were 1,089 DEGs in the RNA-sequencing dataset, including 565 upregulated genes and 524 downregulated genes (Supplementary Figure 1).

### The Co-expression Modules in Glomeruli and Tubulointerstitia

**FIGURE 2:**
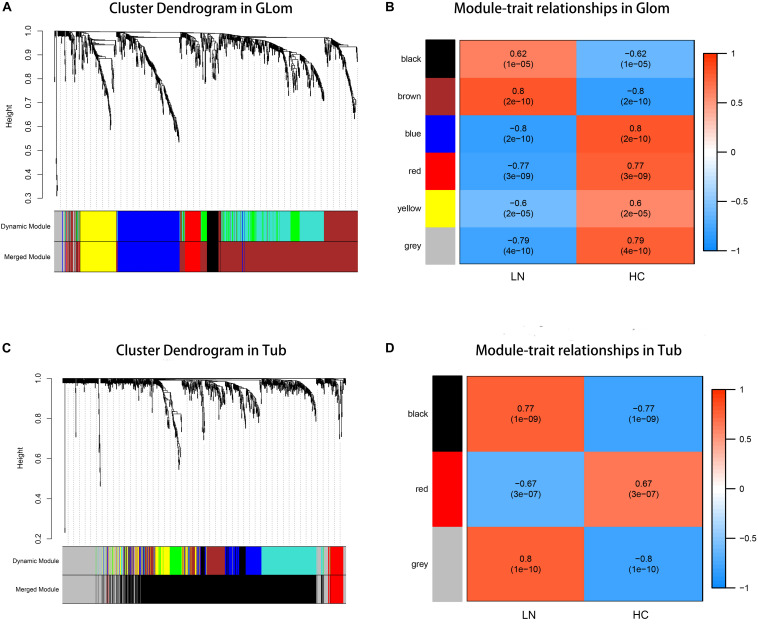
Weighted gene co-expression network analysis (WGCNA) analysis. **(A)** The cluster dendrogram of co-expression genes in glomeruli. **(B)** Module–trait relationships in glomeruli. Each cell contains the corresponding correlation and *p*-value. **(C)** The cluster dendrogram of co-expression genes in tubulointerstitia. **(D)** Module–trait relationships in tubulointerstitia. Each cell contains the corresponding correlation and *p*-value. LN, lupus nephritis; HCs, healthy controls; Glom, glomeruli; Tub, tubulointerstitia.

According to the previously set criteria, there were 998 genes and 955 genes in the glomeruli and tubulointerstitia, respectively, into the WGCNA analysis. With each module assigned a color, a total of five modules were identified in glomeruli (excluding a gray module that was not assigned into any cluster). Then, a heat map was generated regarding module–trait relationships to evaluate the association between each module and two clinical features (LN and control). As shown in Figure 2, The two modules “brown” and “black” were positively associated with LN, and three modules “red,” “yellow,” and “blue” were negatively associated with LN (Figure 2B). Similarly, two modules in tubulointerstitia were identified; the module “brown” was positively associated with LN, and the module “red” was negatively associated with LN (Figure 2D).

### Gene Ontology and Kyoto Encyclopedia of Genes and Genomes Pathway Enrichment Analyses

In glomeruli, the DEGs in the brown module and the black module positively correlated with LN were significantly enriched in immune response, especially against virus infection mediated by type I IFN, such as “response to virus,” “defense response to virus,” and “type I interferon signaling pathway.” The KEGG pathway analysis revealed that the abnormal signaling pathways induced during some infectious diseases, such as those caused by influenza A, herpes simplex, and *Staphylococcus aureus*, were similar to the pathways deployed during the development of LN. The red module was negatively related to LN, and the enrichment analysis showed some biochemical reactions and metabolic pathways are impaired in LN, such as cellular oxidant detoxification, sodium-independent organic anion transport, biosynthesis of amino acids, and protein digestion and absorption. Furthermore, the enrichment analysis for the blue module negatively related to LN also showed the regulation of muscle contraction, response to toxic substances, and Rap1 signaling pathway were also abnormal (Table 1).

**TABLE 1:** GO and KEGG enrichment analysis of DEGs in co-expression modules of glomeruli.

**Modules**	**The number of DEGs**	GO and KEGG terms*	***p*-value**
Brown module	256	GO:0009615: Response to virus	6.99E–25
		GO:0051607: Defense response to virus	1.01E–23
		GO:0060337: Type I interferon signaling pathway	4.68E–19
		GO:0045071: Negative regulation of viral genome replication	1.31E–16
		GO:0045087: Innate immune response	2.14E–15
		hsa05150: *Staphylococcus aureus* infection	2.92E–09
		hsa05164: Influenza A	1.12E–07
		hsa05152: Tuberculosis	4.49E–06
		hsa05133: Pertussis	8.99E–06
		hsa05168: Herpes simplex infection	3.19E–05
Black module	8	GO:0060337: Type I interferon signaling pathway	0.023
		hsa04622: RIG-I-like receptor signaling pathway	0.040
Red module^#^	3	–	–
Yellow module	45	GO:0098869: Cellular oxidant detoxification	6.60E–04
		GO:0043252: Sodium-independent organic anion transport	0.001
		GO:0055114: Oxidation-reduction process	0.003
		GO:0042157: Lipoprotein metabolic process	0.004
		GO:0006094: Gluconeogenesis	0.005
		hsa01100: Metabolic pathways	0.001
		hsa01130: Biosynthesis of antibiotics	0.002
		hsa01230: Biosynthesis of amino acids	0.004
		hsa04974: Protein digestion and absorption	0.007
		hsa00260: Glycine, serine, and threonine metabolism	0.013
Blue module	33	GO:0006937: Regulation of muscle contraction	2.24E–04
		GO:0032972: Regulation of muscle filament sliding speed	0.003
		GO:0009636: Response to toxic substance	0.008
		GO:0055010: Ventricular cardiac muscle tissue morphogenesis	0.040
		GO:0055010: Negative regulation of insulin receptor signaling pathway	0.046
		hsa04015: Rap1 signaling pathway	0.025

*^∗^If GO or KEGG terms were more than five, only the top five terms were displayed. ^#^The red module was not enriched in significant terms. DEGs, differentially expressed genes; GO, Gene Ontology; KEGG, Kyoto Encyclopedia of Genes and Genomes.*

In tubulointerstitia, the black module positively related to LN was enriched in the type I IFN pathway, as in the glomerulus. In the red module negatively related to LN, the enrichment analysis showed the DEGs were mainly enriched in cellular response to hormone stimulus, response to cAMP, transcriptional action, and osteoclast differentiation (Table 2).

**TABLE 2:** GO and KEGG enrichment analysis of DEGs in co-expression modules of tubulointerstitia.

**Modules**	**The number of DEGs**	GO and KEGG terms*	***p*-value**
Black module	106	GO:0060337: Type I interferon signaling pathway	2.92E–34
		GO:0009615: Response to virus	1.24E–21
		GO:0051607: Defense response to virus	2.99E–21
		GO:0006955: Immune response	8.47E–19
		GO:0045071: Negative regulation of viral genome replication	3.31E–18
		hsa05168: Herpes simplex infection	5.31E–15
		hsa05332: Graft vs. host disease	1.61E–14
		hsa05330: Allograft rejection	5.92E–14
		hsa05150: *Staphylococcus aureus* infection	8.92E–14
		hsa04940: Type I diabetes mellitus	2.43E–13
Red module	16	GO:0032870: Cellular response to hormone stimulus	3.23E–10
		GO:0051591: Response to cAMP	6.58E–08
		GO:0006366: Transcription from RNA polymerase II promoter	3.12E–06
		GO:0045944: Positive regulation of transcription from RNA polymerase II promoter	9.67E–06
		GO:0035914: Skeletal muscle cell differentiation	1.04E–05
		hsa05166: HTLV-I infection	3.19E–04
		hsa04380: Osteoclast differentiation	7.34E–04
		hsa05031: Amphetamine addiction	0.004
		hsa04010: MAPK signaling pathway	0.005

*^∗^If GO or KEGG terms were more than five, only the top 5 terms were displayed. DEGs, differentially expressed genes; GO, Gene Ontology; KEGG, Kyoto Encyclopedia of Genes and Genomes; MAPK, mitogen-activated protein kinase.*

### Gene Transcription in Glomeruli and Tubulointerstitia Was Affected by Each Other

**FIGURE 3:**
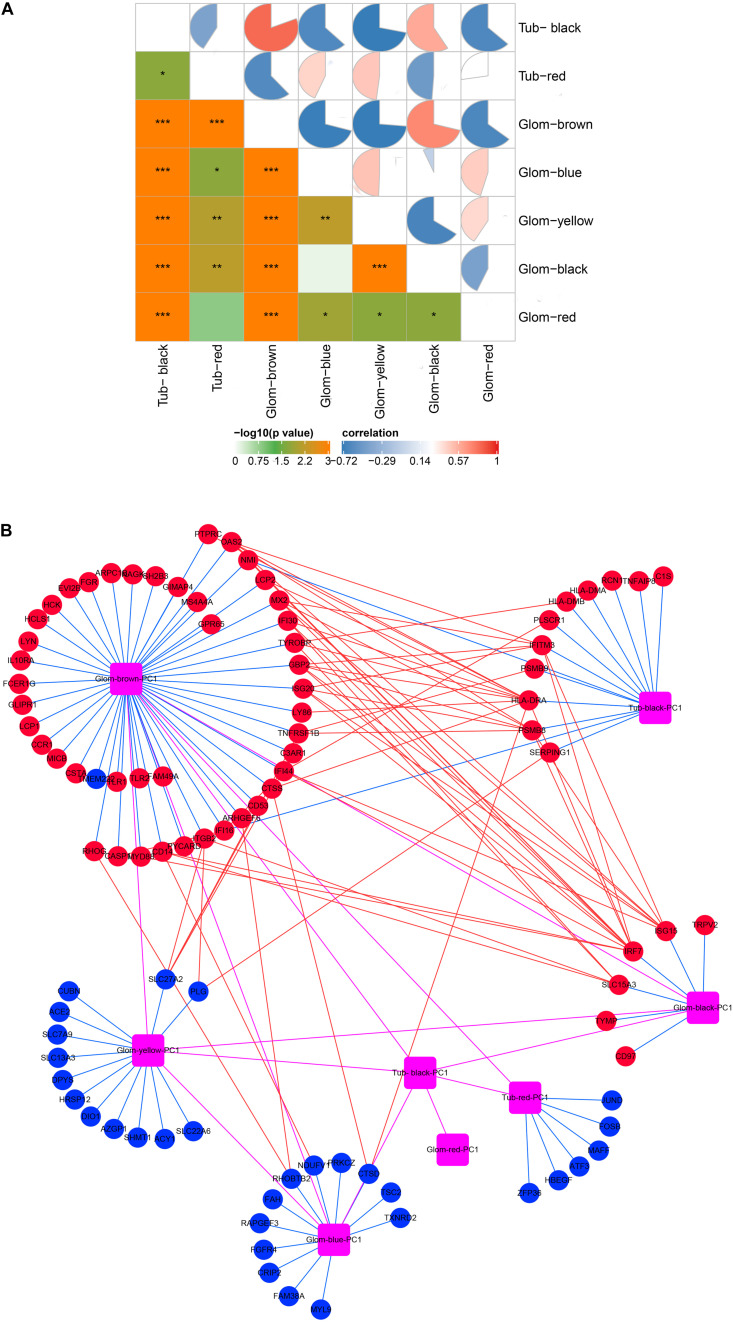
Molecular crosstalk analysis. **(A)** The correlation between modules in glomeruli and tubulointerstitia. **(B)** The interaction of hub genes located in various modules. Glom, glomeruli; Tub, tubulointerstitia; PC1, first principal component. Blue lines represent inclusion relationship of modules to hub genes; red lines represent the interaction between the hub genes located in different modules; magenta lines represent the interaction between various modules; red represents upregulation; blue represents downregulation. “*” represents *p* < 0.05, “**” represents *p* < 0.01, “***” represents *p* < 0.001.

As shown in Figure 3A, there was a high correlation between the various modules. The black module in the tubulointerstitia had different effects on almost every module in the glomeruli. Positive correlation in the glomeruli was found with the brown module and the black module, but negative correlation with the blue, yellow, and red modules. Similarly, the brown module in glomeruli is positively correlated with the black module but negatively correlated with the red module in the tubulointerstitia. The strong correlation between these modules suggested that the genes transcribed in glomeruli and tubulointerstitia may interact with each other. The PPI network between these modules suggested some hub genes acted as bridges between these modules (Figure 3B). We calculated the degree of each hub gene using the “Network analysis” tool in Cytoscape 3.7.2. The top 10 genes with the highest degrees were obtained, including *IRF7*, *HLA-DRA*, *ISG15*, *PSMB8*, *IFITM3*, *GBP2*, *OAS2*, *SLC27A2*, *SLC15A3*, and *IFI44*; hence, these genes were defined as crosstalk genes.

### Performance of Immune Cell Infiltration Analysis

**FIGURE 4:**
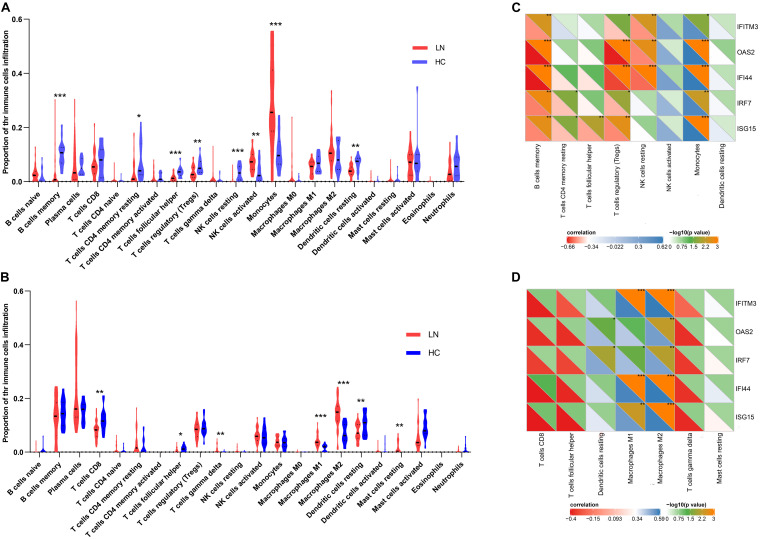
Immune cell infiltration analysis. **(A)** The proportion of the immune cell infiltration in glomeruli. **(B)** The proportion of the immune cell infiltration in tubulointerstitia. **(C)** The correlation between the crosstalk genes and eight types of immune cells in glomeruli. **(D)** The correlation between the crosstalk genes and seven types immune cells in tubulointerstitia. “*” represents *p* < 0.05, “**” represents *p* < 0.01, “***” represents *p* < 0.001.

As mentioned above, the type I IFN response was very significant in LN. Considering that some immune cells play salient roles in the type I IFN response, we used the CIBERSORT algorithm to estimate the proportion of various types of immune cells in the kidney and explore their relationship with IFN-induced genes. The results showed that the number of monocytes increased significantly in the glomeruli of the LN group compared with that in the control. Moreover, the number of activated NK cells was also increased. On the contrary, the number of memory B cells, T follicular helper cells (Tfh cells), T regulatory cells (Tregs), resting NK cells, resting dendritic cells, and resting memory CD4 T cells was decreased (Figure 4A). In the tubulointerstitia, the number of M1 and M2 macrophages, gamma delta T cells, and resting mast cells was increased, whereas that of CD8 T cells, Tfh cells, and resting dendritic cells was decreased (Figure 4B).

The Pearson correlation analysis showed that the IFN-induced genes, *IRF7*, *ISG15*, *IFITM3*, *OAS2*, and *IFI44*, in the crosstalk gene set were associated with immune infiltration. In glomeruli, these hub genes were positively correlated with monocytes but negatively correlated with memory B cells and Tregs (Figure 4C). In the tubulointerstitia, the IFN-induced genes were positively correlated with M1 and M2 macrophages (Figure 4D).

### Validation of Crosstalk Genes by Next-Generation Sequencing

To verify our analysis, we extracted the expression level of these crosstalk genes using NGS and found that most crosstalk genes had the same changes in the RNA-sequencing dataset (Table 3), illustrating a satisfactory reliability of the result. The expression levels of *HLA-DRA*, *GBP2*, and *SLC27A2* did not differ in our sequencing (*p* > 0.05), but they showed the same trends as microarray sequencing. In the future, we will expand the sample size to validate these crosstalk genes.

**TABLE 3:** The FC value of crosstalk genes in GSE32591 and RNA-sequencing dataset.

	**Glom**		**Tub**		**Kidney**	
	**FC**	***p*-value**	**FC**	***p*-value**	**FC**	***p*−value**
*IRF7*	3.138	< 0.001	1.579	< 0.001	1.989	0.004
*HLA-DRA*	1.876	< 0.001	2.152	< 0.001	1.015	0.915
*ISG15*	6.561	< 0.001	9.980	< 0.001	5.732	< 0.001
*PSMB8*	1.543	0.004	2.836	< 0.001	1.780	< 0.001
*IFITM3*	2.530	< 0.001	3.278	< 0.001	1.707	0.004
*GBP2*	3.706	< 0.001	1.509	0.007	1.042	0.878
*OAS2*	5.979	< 0.001	1.911	< 0.001	3.621	< 0.001
*SLC27A2*	0.401	< 0.001	0.988	0.895	0.655	0.607
*SLC15A3*	2.359	< 0.001	1.173	< 0.001	2.228	< 0.001
*IFI44*	9.088	< 0.001	7.989	< 0.001	3.997	< 0.001

*FC, fold change; Glom, glomeruli; Tub, tubulointerstitia.*

### Candidate Lupus Nephritis-Related Small Molecular Compounds

**FIGURE 5:**
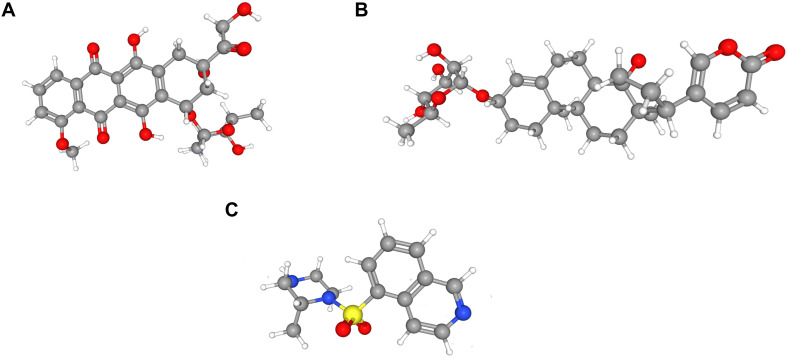
Three-dimensional (3D) chemical structures of the three molecules. **(A)** Doxorubicin. **(B)** Proscillaridin. **(C)** H-7.

To identify LN-related small molecular compounds accurately, we integrated the DEGs between GSE32591 and RNA-sequencing dataset and obtained 50 common DEGs, including 38 upregulated genes and 12 downregulated genes (Table 4). Most of the common DEGs were IFN-induced genes, and their biological processes are mainly related to type I IFN signaling pathway (Supplementary Figure 2). Then, we queried the CMap database using the upregulated and downregulated genes and identified some compounds that might influence LN; the 10 compounds are shown in Table 5. Doxorubicin and H-7 were the first two small-molecule drugs with the highest enrichment score, and proscillaridin was the small molecular drug with the lowest enrichment scores; their 3D chemical structures were also downloaded from Pubchem database (Figures 5A–C).

**TABLE 4:** The common DEGs in GSE32591 dataset and RNA-sequencing dataset.

**Expression**	**Genes**
Up	*STAT1, IFI44L, MX1, IFI44, RSAD2, IFI6, MX2, HERC6, ISG15, OAS2, OAS3, OAS1, HERC5, XAF1, IFI27, IFIT1, IFITM1, IFIT3, PARP12, SAMSN1, RTP4, HLA-DQA1, NNMT, PTGER2, LTF, SRGN, PSMB9, TFPI2, SLC15A3, UCP2, ARPC1B, DDX60, LY6E, BST2, MMP7, CFB, UBE2L6, CLU*
Down	*ATF3, EGR1, ZFPM2, FOS, EGR3, CHI3L1, MYL9, TNNC1, FOSB, JUNB, JUN, ZFP36*

*DEGs, differentially expressed genes.*

**TABLE 5:** Ten small molecular compounds for lupus nephritis obtained from the Connectivity Map (CMap) database.

**Rank**	**CMap name**	***n***	**Enrichment**	***p***
1	Geldanamycin	15	0.661	0
2	Tanespimycin	62	0.583	0
3	Proscillaridin	3	–0.983	0.00002
4	H-7	4	0.922	0.00004
5	Lisuride	5	–0.807	0.00062
6	5155877	4	–0.860	0.00068
7	Meclocycline	4	–0.859	0.00068
8	Doxorubicin	3	0.921	0.00106
9	Lycorine	5	–0.775	0.00106
10	Lomustine	4	–0.819	0.00203

### Targeting Association Between Signal Transducer and Activator of Transcription 1 and Proscillaridin via Molecular Docking

**FIGURE 6:**
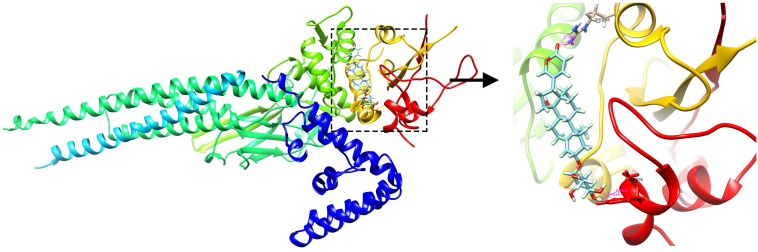
The docking simulation result showing hydrogen bonding between proscillaridin and the ARG586, HSD675, and ALA676 residues in the SH2 domain of signal transducer and activator of transcription 1 (STAT1).

Proscillaridin was reported to inhibit signal transducer and activator of transcription (STAT)3, and the protein STAT1 encoded by the upregulated DEG *STAT1* has been shown to have a structure similar to that of STAT3. We speculated that proscillaridin could also inhibit STAT1. Molecular docking was performed to preliminarily verify whether there is direct targeting between compounds and the protein. The results showed that the ARG586, HSD675, and ALA676 residues form hydrogen bonds with proscillaridin, which indicated that proscillaridin mainly interacts with the SH2 domain of STAT1 (Figure 6).

## Discussion

In recent years, with the wide use of immunosuppressants and biological agents, the prognosis and survival rate of patients with LN have improved; however, 10–20% of the patients with LN progress to ESRD, which is linked to a heavy burden and morbidity (Aljaberi et al., 2019). So, there is a need to study the pathophysiology and discover new therapeutic methods to prevent LN progression and prolong patient survival. Therefore, we performed sequencing in LN renal tissues and healthy renal tissues to identify DEGs and explore their roles in LN.

Through GO and KEGG pathway enrichment analyses of DEGs, we found that innate and adaptive immune response, especially against virus infection mediated by type I IFN, was highly active in both glomeruli and tubulointerstitia, such as the brown module and the black module in glomeruli and the black module in tubulointerstitia. Besides, the results also showed that the metabolism process of carbohydrate, protein, and lipid in LN patients was disordered, and some biochemical reactions involving detoxification were impaired. Interestingly, we found the blue module in glomeruli was enriched in the regulation of muscle contraction, which indicated that the contraction of mesangial cells (Jankowski et al., 2003), podocytes (Saleem et al., 2008), and capillaries might be dysregulated. This may lead to a decrease of the glomerular filtration rate (GFR) and might be one of the causes of urine protein in LN patients (Stockand and Sansom, 1998). In the tubulointerstitia, the red module was enriched in response to hormone stimulus and cAMP. Many types of ion transport are mediated via cAMP, such as Na^+^, K^+^, Ca^2+^, and Cl^–^ (Li et al., 2008). The dysregulation might affect the tubules, then the filtration and reabsorption of tubules would be impaired in LN patients.

Glomerular lesions and tubulointerstitial lesions often occurred together in LN (Cimbaluk and Naumann, 2017), so we wanted to explore whether the two lesions were related at the genetic level. Therefore, we further used PCA and correlation analysis to explore the interaction between glomeruli and tubulointerstitial modules. There was a high correlation between the various modules that suggested that the gene transcription in glomeruli and tubulointerstitia may interact with each other. Combined with gene enrichment results, clearly, the high IFN response in glomeruli and tubulointerstitia revealed a mutual promotion. For a long time, we focused on the fact that IFN could result in autoimmune inflammation in LN (Eloranta et al., 2013); however, our molecular crosstalk analysis showed that the IFN response might also affect some biological processes, such as metabolic pathways, muscle contraction, and detoxification process in glomeruli. In the tubulointerstitia, the cellular response to hormone stimulus and cAMP and transcriptional activation were highly negatively correlated with IFN response, which indicated that the IFN response might have adverse effects on these biological processes in the tubulointerstitia. Most crosstalk genes interpreted from the PPI analysis were IFN-induced genes, which also indicated that IFN-induced genes played an important role in the transcription of each module. Except the IFN-induced genes, we also found some new genes, such as *SLC27A2*, *SLC15A3*, *HLA-DRA*, and *PSMB8*, which also might be important in kidney gene transcription.

To further explore the relationship between type I IFN response and immune cells in kidney, immune cell infiltration analysis was performed, and the results showed monocytes were the prominent differentially expressed cells in glomeruli and were positively correlated with IFN-induced genes. Monocytes are important subsets of immune cells, participate in various types of immune responses, thereby playing an important role in autoimmune diseases (Auffray et al., 2009). Uccellini and García-Sastre (2018) observed high IFN response in inflammatory monocytes during infection. Monocytes also have been reported to produce IFN and mediate tissue damage in H1N1 IAV-infected mouse models (Lin et al., 2014). Therefore, we speculated that there might be a mutual promotion between the monocytes and the high IFN response in glomeruli. However, we found that these IFN-induced genes seemed to be negatively correlated with Tregs and memory B cells. The function of Tregs is that they suppress autoreactive lymphocytes, especially CD8^+^ T cell and B cell activation, and maintain self-tolerance (Ohl and Tenbrock, 2015). It has been reported that the defects in Tregs or a lack of Tregs is associated with SLE pathogenesis (Ohl and Tenbrock, 2015). So we speculated that the decrease of Tregs in LN leads to the weakening of the inhibitory effect on B cells, thereby enhancing the B cell intrinsic effect for the augmentation of IFN. Besides, the reduction of memory B cells caused by the disturbance of B cell homeostasis has been observed in active SLE (Odendahl et al., 2000). We speculated that the decreased memory B cells might be related to the abnormal activation of B cells. The activated B cells circulate in the peripheral blood and participate in the formation of autoantibodies and IFN response (Eloranta et al., 2013). Therefore, there is a negative correlation between memory B cells and IFN-induced genes. Macrophages were found to be mainly elevated in the tubulointerstitia and positively correlated with IFN-induced genes. There are two major polarization states for macrophages; “M1” macrophages produce a lot of pro-inflammatory cytokines including IFN-α to cause tissue damage. On the contrary, “M2”-type macrophages can repair tissue damage by secreting anti-inflammatory cytokines such as IL-10 and CCL18 (Wen et al., 2019). The increased numbers of M1 and M2 macrophages will cause repeated injury and repair of the tubulointerstitia, leading to the fibrosis of tubulointerstitia.

Through CMap analysis, 10 drugs (geldanamycin, tanespimycin, proscillaridin, H-7, lisuride, 5155877, meclocycline, doxorubicin, lycorine, lomustine) were identified that might induce the development of LN (enrichment score > 0) or which may be potential drugs for the treatment of LN (enrichment factor < 0). Doxorubicin and H-7 were the first two small-molecule drugs with the highest enrichment scores, which indicated that the use of these small molecules or their analogs might induce or aggravate LN. Huang et al. (2004) reported a patient with SLE developing lupus-like symptoms, such as fever, erythema, and exfoliative dermatitis, with a positive lupus band test after using doxorubicin. Yang et al. (2009) found that doxorubicin treatment in mice significantly increased albuminuria and decreased podocytes. These results showed that patients with LN should be cautious when using doxorubicin. H-7 is a protein kinase inhibitor (Steele and Brahmi, 1988) and has not been reported to be associated with LN. Proscillaridin was the first small molecular drugs with the lowest enrichment score, indicating that it might be a potential therapeutic strategy for LN. In short, the abovementioned drugs might affect LN through a variety of small molecular pathways.

The DEG *STAT1* was upregulated in the common DEGs (Table 2). STAT1 is known to occupy a central position in the type I IFN signaling pathway. If drugs that can inhibit STAT1 and change the high IFN-response signature are identified, they may be considered as potential candidate drugs for LN treatment. Proscillaridin belongs to cardiac glycosides (Maryam et al., 2018), and Ye et al. (2011) have reported that cardiac glycosides could potently inhibit the induction of the IFN genes induced by virus, double-stranded RNA, and double-stranded DNA, which was consistent with our analysis. Proscillaridin was also reported to have an inhibitory effect on STAT3 (Maryam et al., 2018). As STAT1 and STAT3 belong to the STAT protein family and have similar structures, and proscillaridin reverses the high IFN-response signature, we speculated that it could also inhibit STAT1. Through molecular docking, we found that proscillaridin formed four hydrogen bonds with the SH2 domain of STAT1. The SH2 domain is the most critical and conserved domain in STAT1, located between amino acid residues 577 and 683; it is vital for the activation and function of STAT1 (Levy and Darnell, 2002). Proscillaridin might inhibit the activation of STAT1 and the type I IFN signaling pathway by binding to the SH2 domain. However, more details of the specific interactions between proscillaridin and STAT1 need to be confirmed by future experiments.

However, there remain several limitations that need to be resolved in the future. For example, our research was a bioinformatic analysis based on sequencing data; therefore, further verifications by cell and animal experiments are needed. Besides, whether the small molecular compounds screened in our study could influence LN and the specific interactions and mechanisms between proscillaridin and STAT1 need further confirmation. Next, better-designed experiments need to be carried out based on our findings.

In conclusion, we found that type I IFN response was highly active, and some biological processes such as metabolism, detoxification, ion transport were impaired in LN through the WGCNA analysis of DEGs. The gene transcription in glomeruli and tubulointerstitia might affect each other, and some crosstalk genes, such as *IRF7*, *HLA-DRA*, *ISG15*, *SLC15A3*, and *IFITM3*, play important roles in this process. Monocytes and macrophages may be associated with high IFN response in kidney tissues. Proscillaridin may play a therapeutic role by targeting STAT1. Therefore, the analysis for DEGs provided a new perspective for the pathophysiology and treatment of LN.

## Data Availability Statement

The original sequencing data has been deposited in SRA. To review GEO accession GSE157293: Go to https://www.ncbi.nlm.nih.gov/geo/query/acc.cgi?acc=GSE157293.

## Ethics Statement

The studies involving human participants were reviewed and approved by the Ethics Committee of the First Affiliated Hospital of Zhengzhou University. The patients/participants provided their written informed consent to participate in this study.

## Author Contributions

MY and CG participated in kidney sample collection, data analysis, and manuscript writing. ZZ designed and conducted the whole experiment and finalized the manuscript. CZ and XD participated in data analysis. WS, WL, and QW collected kidney tissues from patients. All authors discussed the outline and commented on the manuscript.

## Conflict of Interest

The authors declare that the research was conducted in the absence of any commercial or financial relationships that could be construed as a potential conflict of interest.

## Footnotes

1 https://david.ncifcrf.gov/

2 http://cibersort.stanford.edu/

3 https://www.affymetrix.com

## Supplementary Material

Supplementary Figure 1 The volcano maps of DEGs in the RNA-sequencing dataset.

Supplementary Figure 2 The GO enrichment analysis of the common DEGs. The number represents the gene counts in the GO terms.
