# Supplementary figures and images for: Identification of Molecular Markers Associated With the Pathophysiology and Treatment of Lupus Nephritis Based on Integrated Transcriptome Analysis
Source: Front Genet. 2020 Dec 15;11:583629. doi: 10.3389/fgene.2020.583629 (PMC7770169; doi:10.3389/fgene.2020.583629)

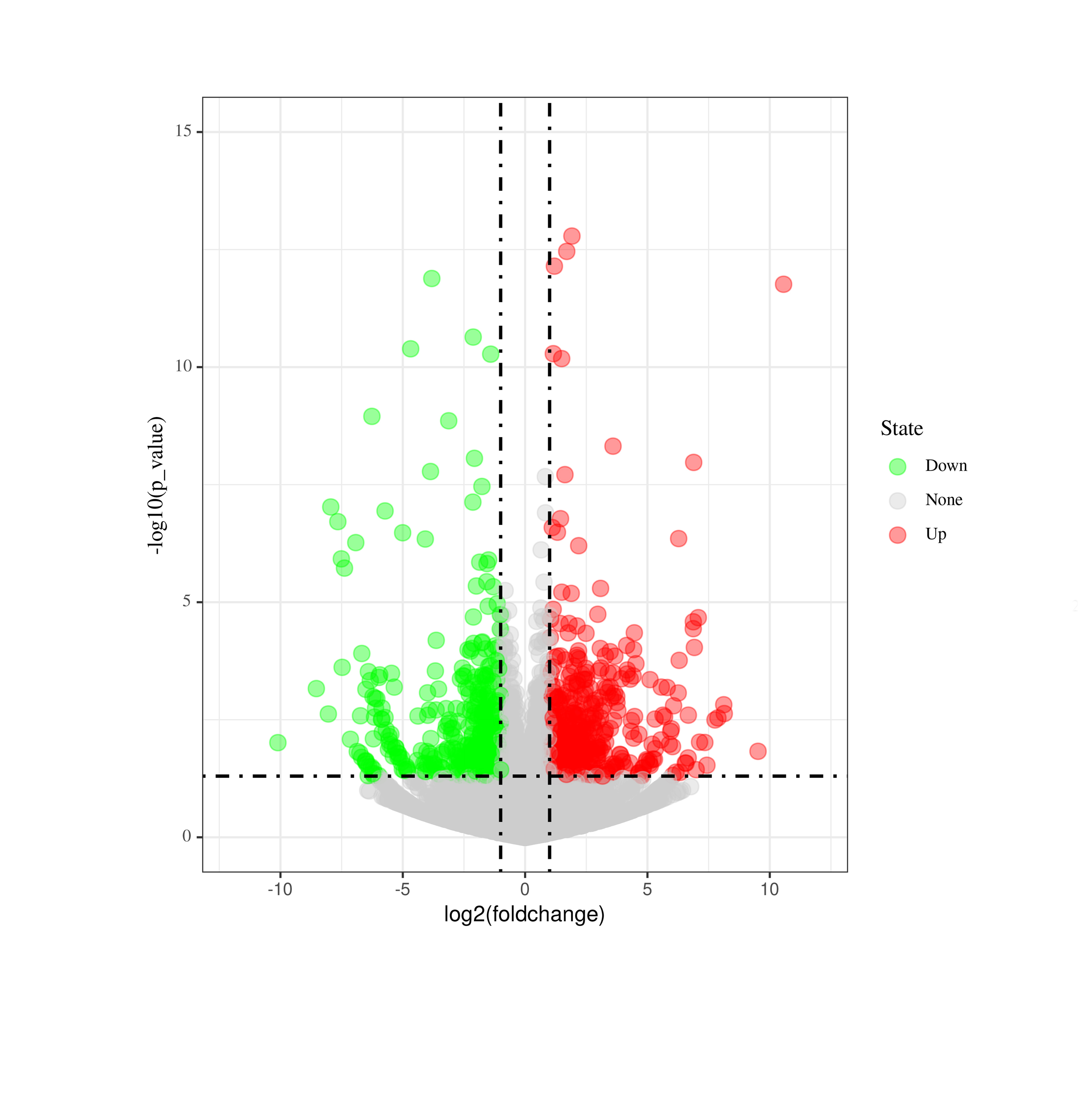

Supplement: Supplementary Figure 1 — The volcano maps of DEGs in the RNA-sequencing dataset. [file Image_1.JPEG]

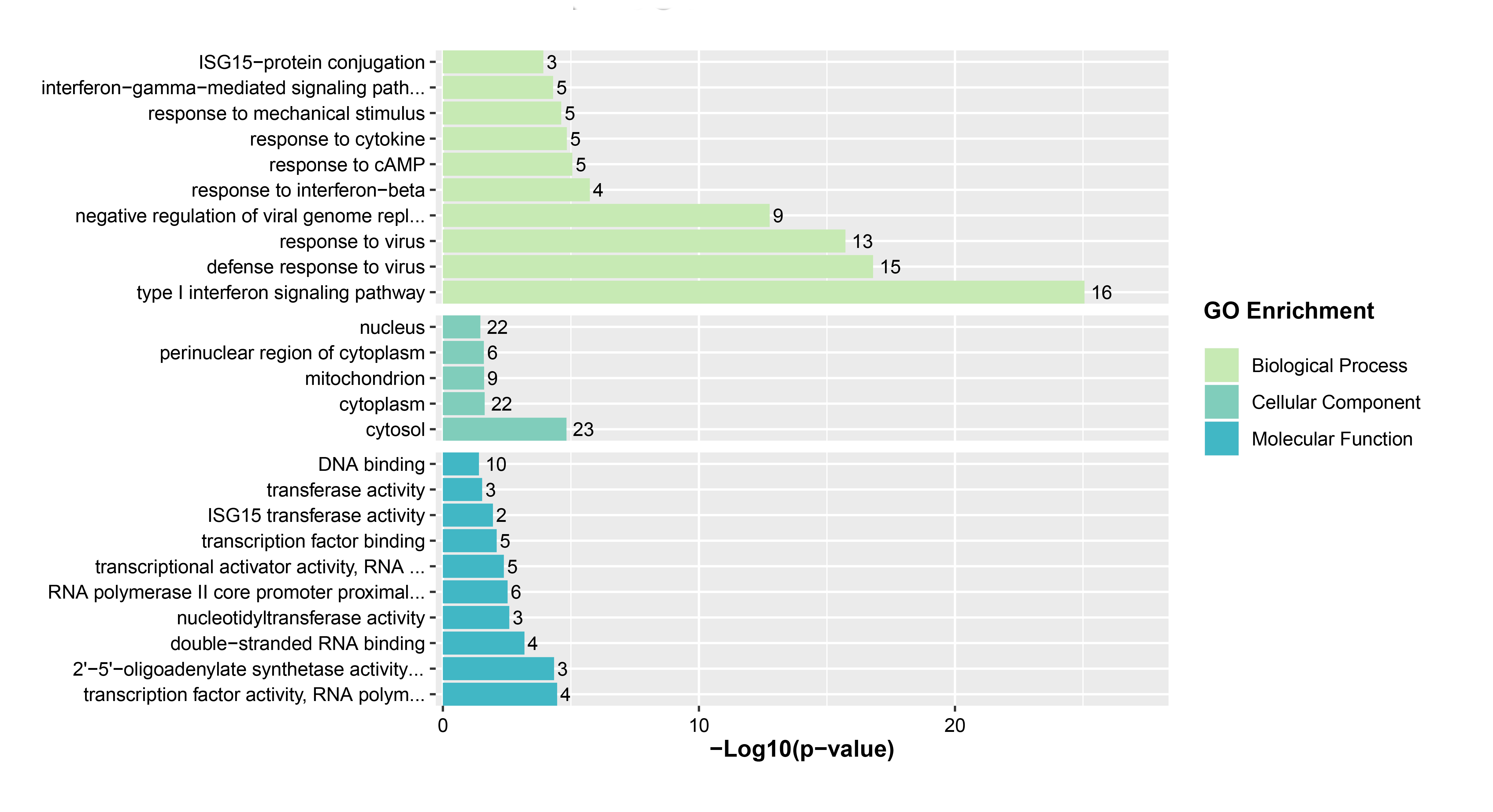

Supplement: Supplementary Figure 2 — The GO enrichment analysis of the common DEGs. The number represents the gene counts in the GO terms. [file Image_2.JPEG]
